# Supplementary material for: Revealing the Drivers Underlying Distinct Evolutionary Trajectories in Lung Adenocarcinoma
Source: bioRxiv. 2025 Dec 23:2025.12.19.695410. Preprint. [Version 1] doi: 10.64898/2025.12.19.695410 (PMC12776081; doi:10.64898/2025.12.19.695410)
Supplement: Supplement 2 [file NIHPP2025.12.19.695410v1-supplement-2.pdf]

bioRxiv preprint doi: https://doi.org/10.64898/2025.12.19.695410; this version posted December 23, 2025. The copyright holder for this preprint (which was not certified by peer review) is the author/funder, who has granted bioRxiv a license to display the preprint in perpetuity. It is made available under aCC-BY 4.0 International license.

# Supplementary Data

**Tables S1-S18**

See accompanying document

**Figure S1: Selection of the optimal number of trajectories**

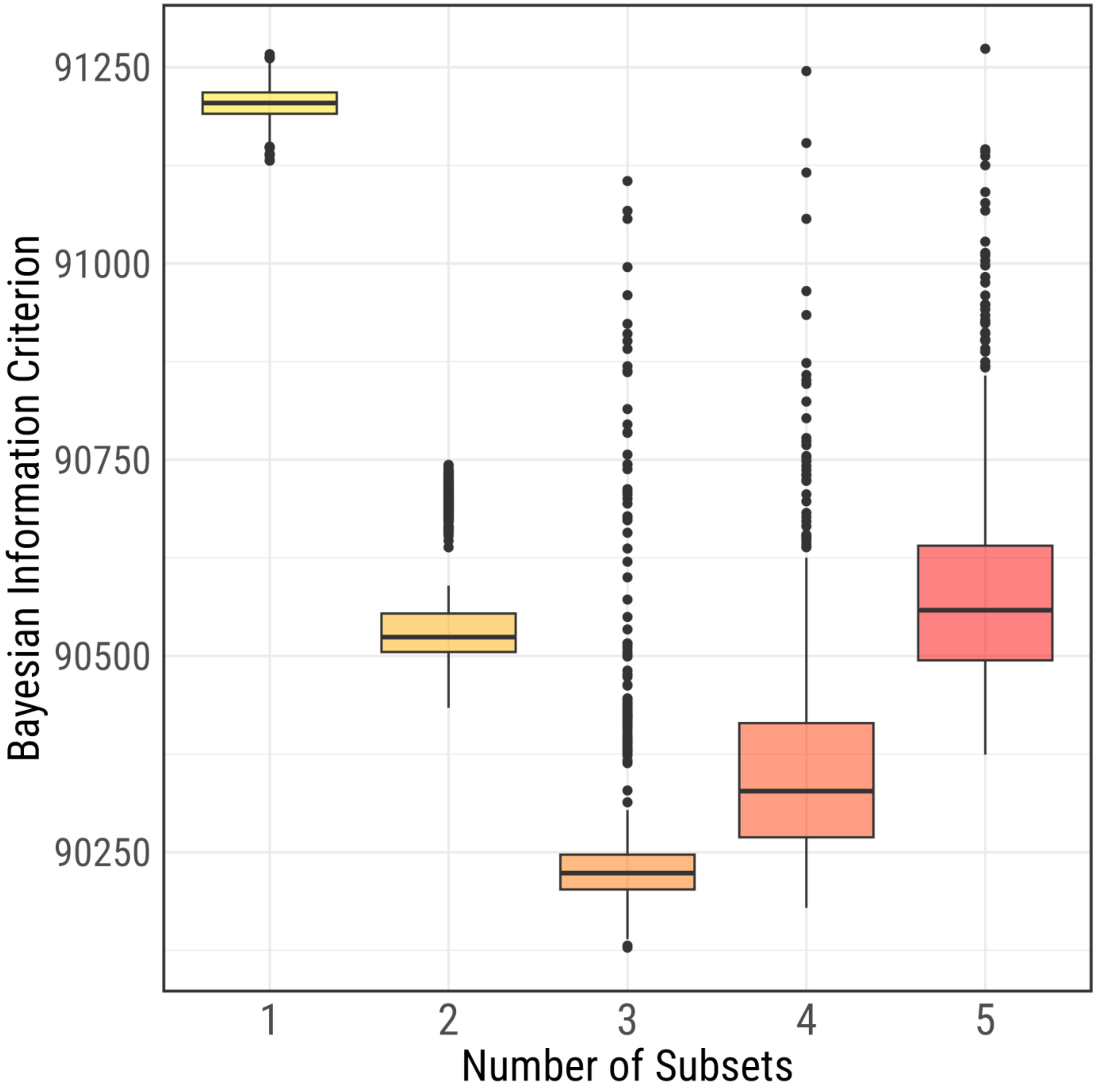

bioRxiv preprint doi: https://doi.org/10.64898/2025.12.19.695410; this version posted December 23, 2025. The copyright holder for this preprint (which was not certified by peer review) is the author/funder, who has granted bioRxiv a license to display the preprint in perpetuity. It is made available under aCC-BY 4.0 International license.

# Figure S2: Event co-occurrence and mutual exclusivity

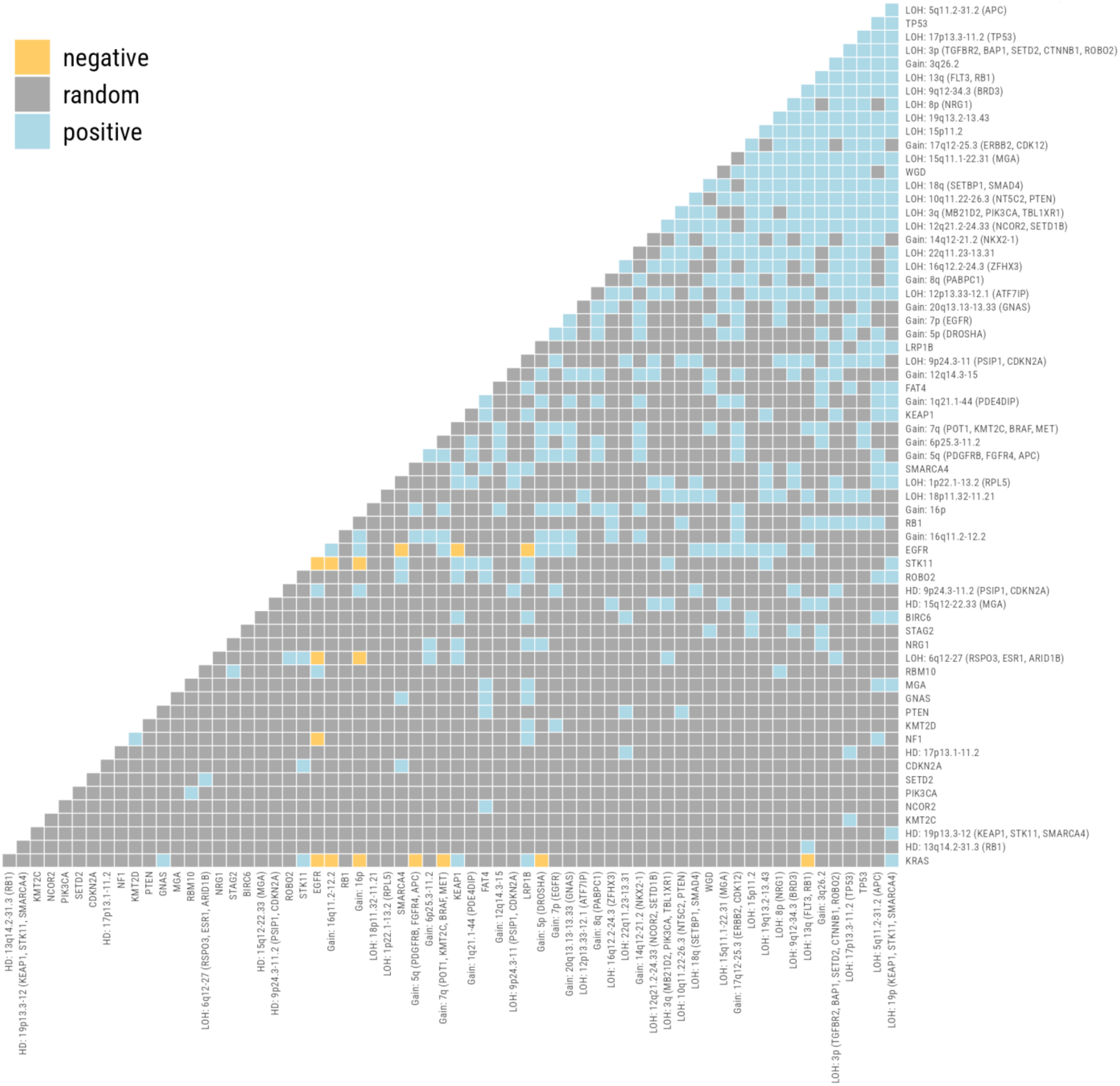

bioRxiv preprint doi: https://doi.org/10.64898/2025.12.19.695410; this version posted December 23, 2025. The copyright holder for this preprint (which was not certified by peer review) is the author/funder, who has granted bioRxiv a license to display the preprint in perpetuity. It is made available under aCC-BY 4.0 International license.

## Figure S3: Demographic distribution across trajectories

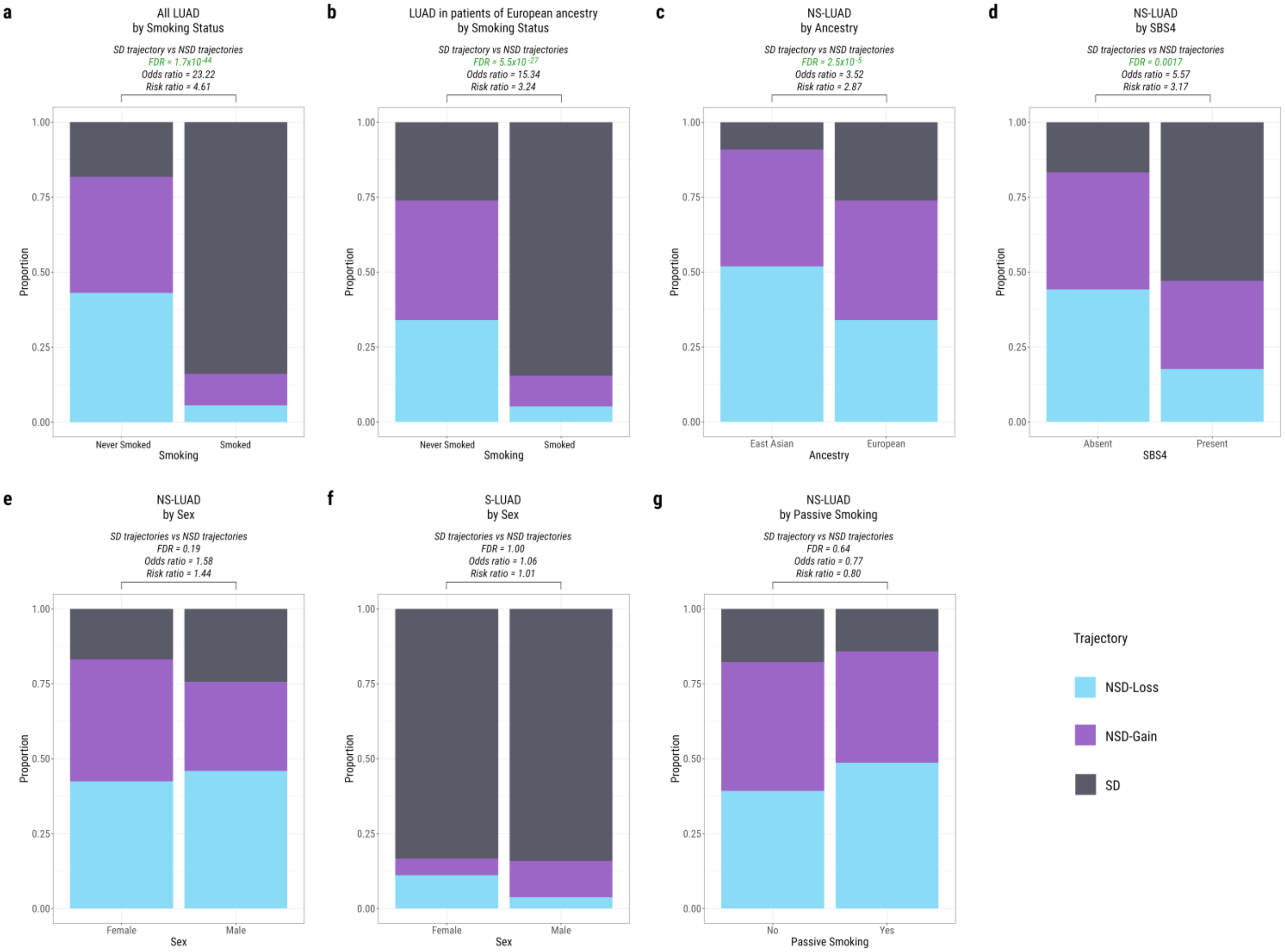

bioRxiv preprint doi: https://doi.org/10.64898/2025.12.19.695410; this version posted December 23, 2025. The copyright holder for this preprint (which was not certified by peer review) is the author/funder, who has granted bioRxiv a license to display the preprint in perpetuity. It is made available under aCC-BY 4.0 International license.

**Figure S4: Events showing a significant preference between S-LUAD and NS-LUAD**

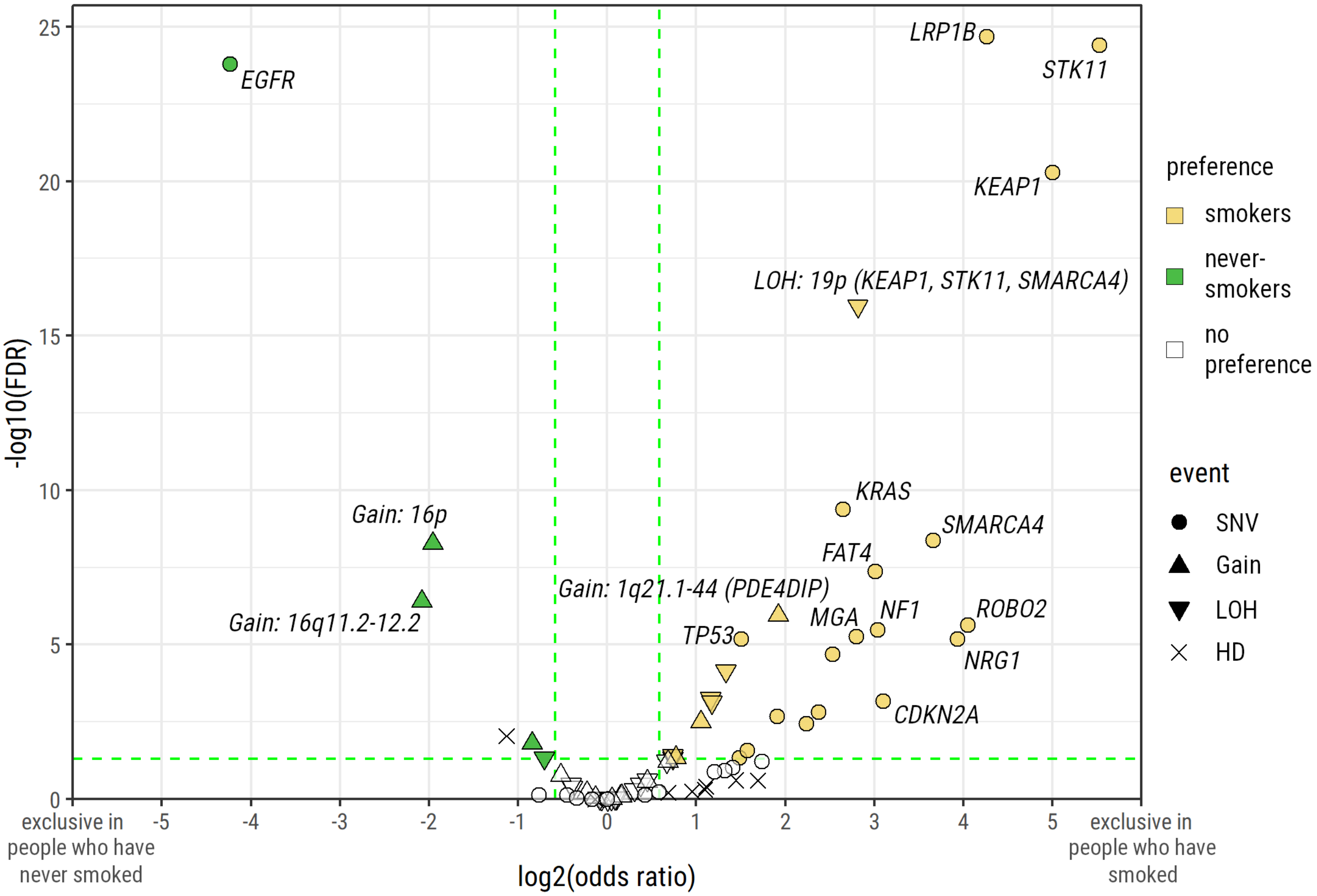

bioRxiv preprint doi: https://doi.org/10.64898/2025.12.19.695410; this version posted December 23, 2025. The copyright holder for this preprint (which was not certified by peer review) is the author/funder, who has granted bioRxiv a license to display the preprint in perpetuity. It is made available under aCC-BY 4.0 International license.

**Figure S5: Major copy number of gains covering the MDM2 locus across different trajectories**

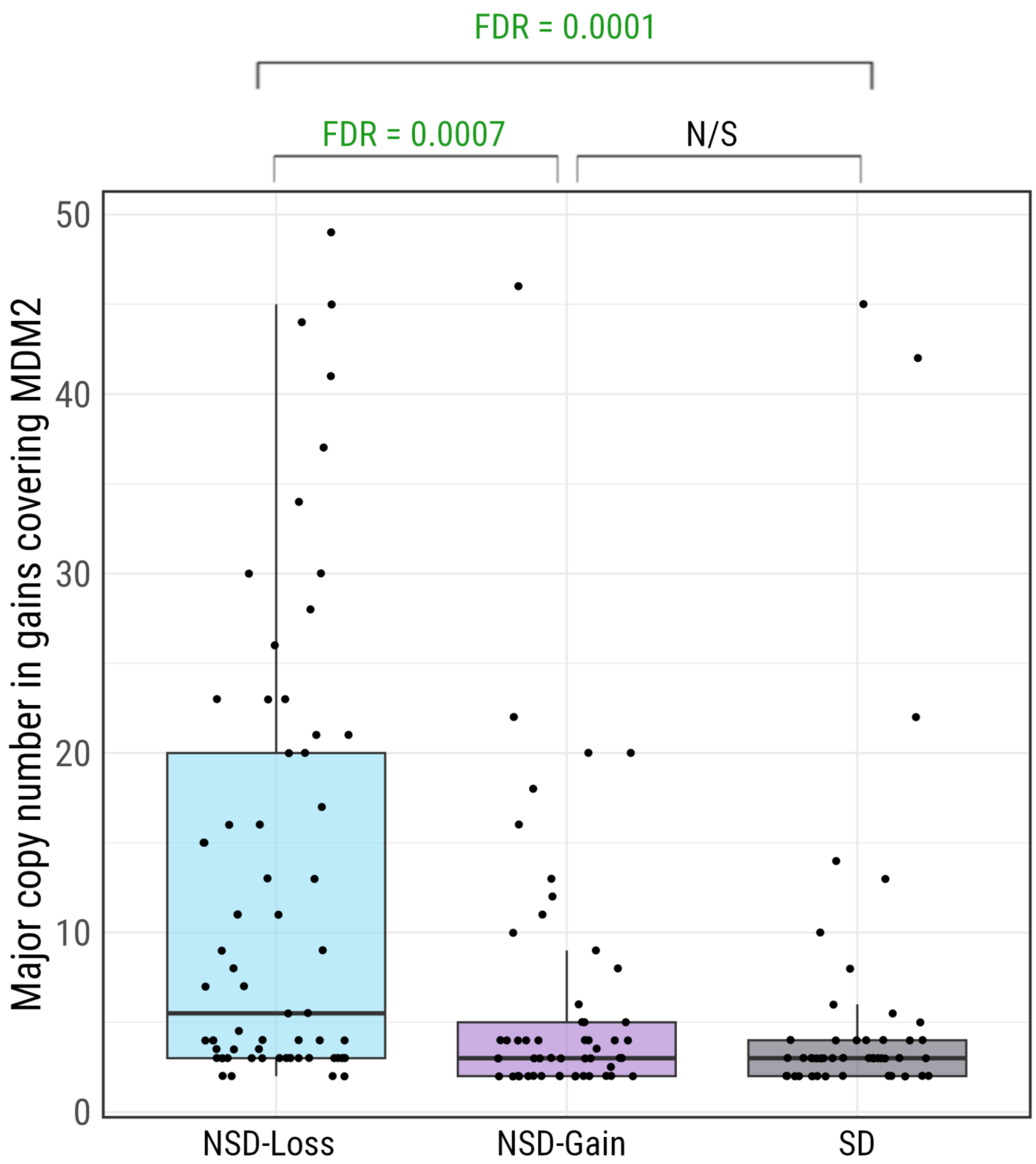

bioRxiv preprint doi: https://doi.org/10.64898/2025.12.19.695410; this version posted December 23, 2025. The copyright holder for this preprint (which was not certified by peer review) is the author/funder, who has granted bioRxiv a license to display the preprint in perpetuity. It is made available under aCC-BY 4.0 International license.

## Figure S6: Mutational signatures showing a significant preference between trajectories

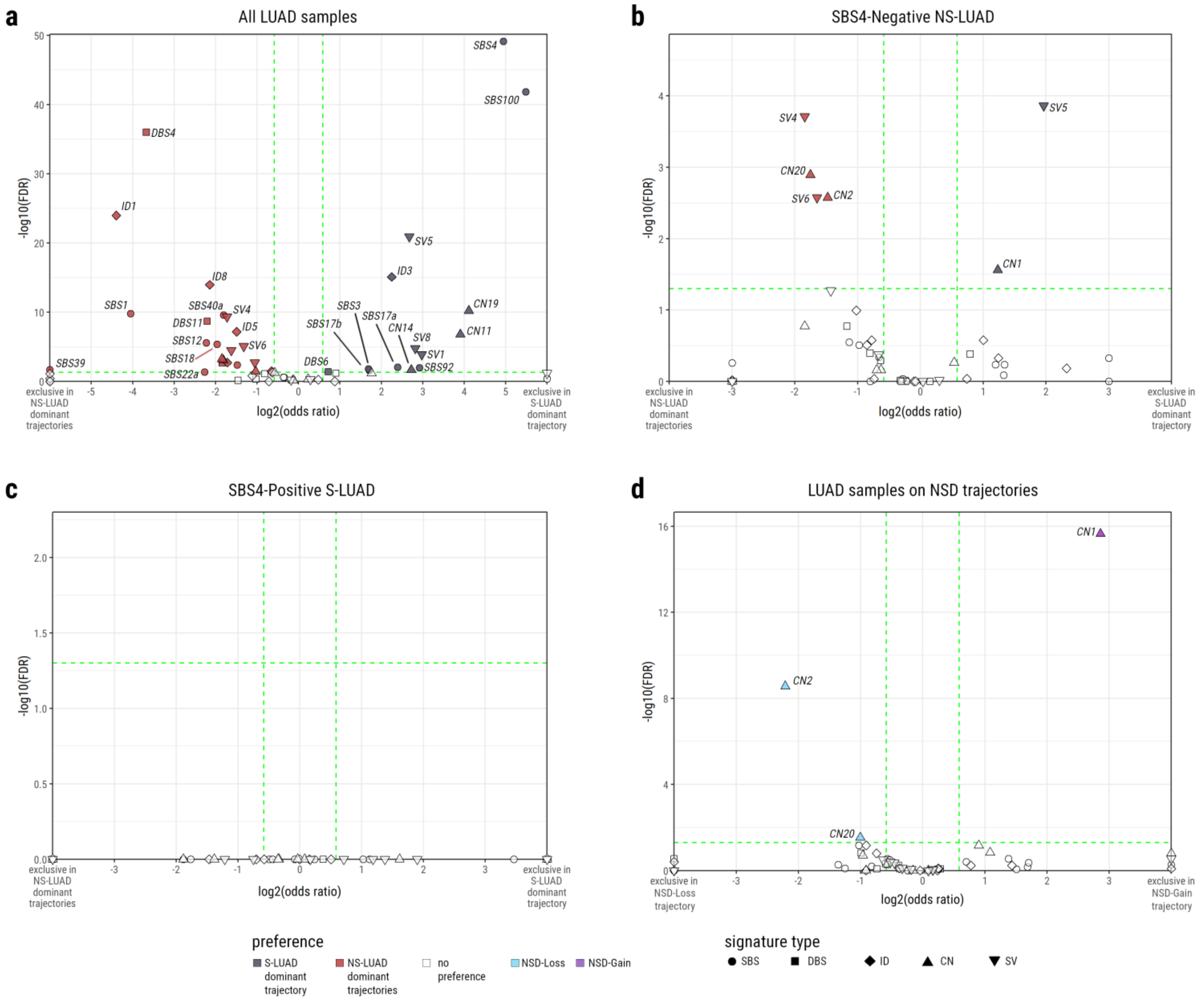

bioRxiv preprint doi: https://doi.org/10.64898/2025.12.19.695410; this version posted December 23, 2025. The copyright holder for this preprint (which was not certified by peer review) is the author/funder, who has granted bioRxiv a license to display the preprint in perpetuity. It is made available under aCC-BY 4.0 International license.

**Figure S7: Adjusted survival curves by trajectory and smoking status**

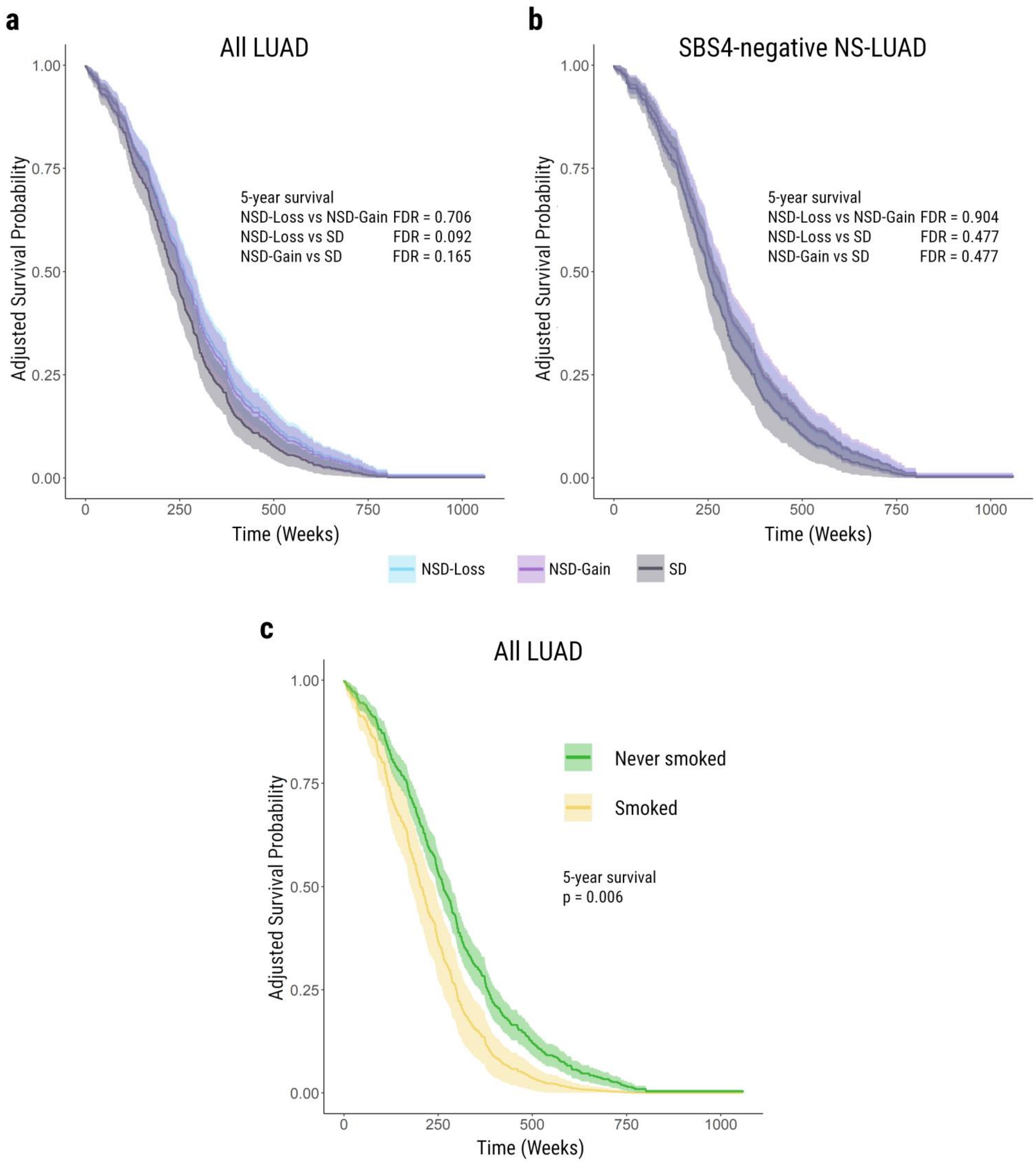

bioRxiv preprint doi: https://doi.org/10.64898/2025.12.19.695410; this version posted December 23, 2025. The copyright holder for this preprint (which was not certified by peer review) is the author/funder, who has granted bioRxiv a license to display the preprint in perpetuity. It is made available under aCC-BY 4.0 International license.

**Figure S8: NS-LUAD event preferences in the absence of *EGFR*, *KRAS*, or *STK11* mutations**

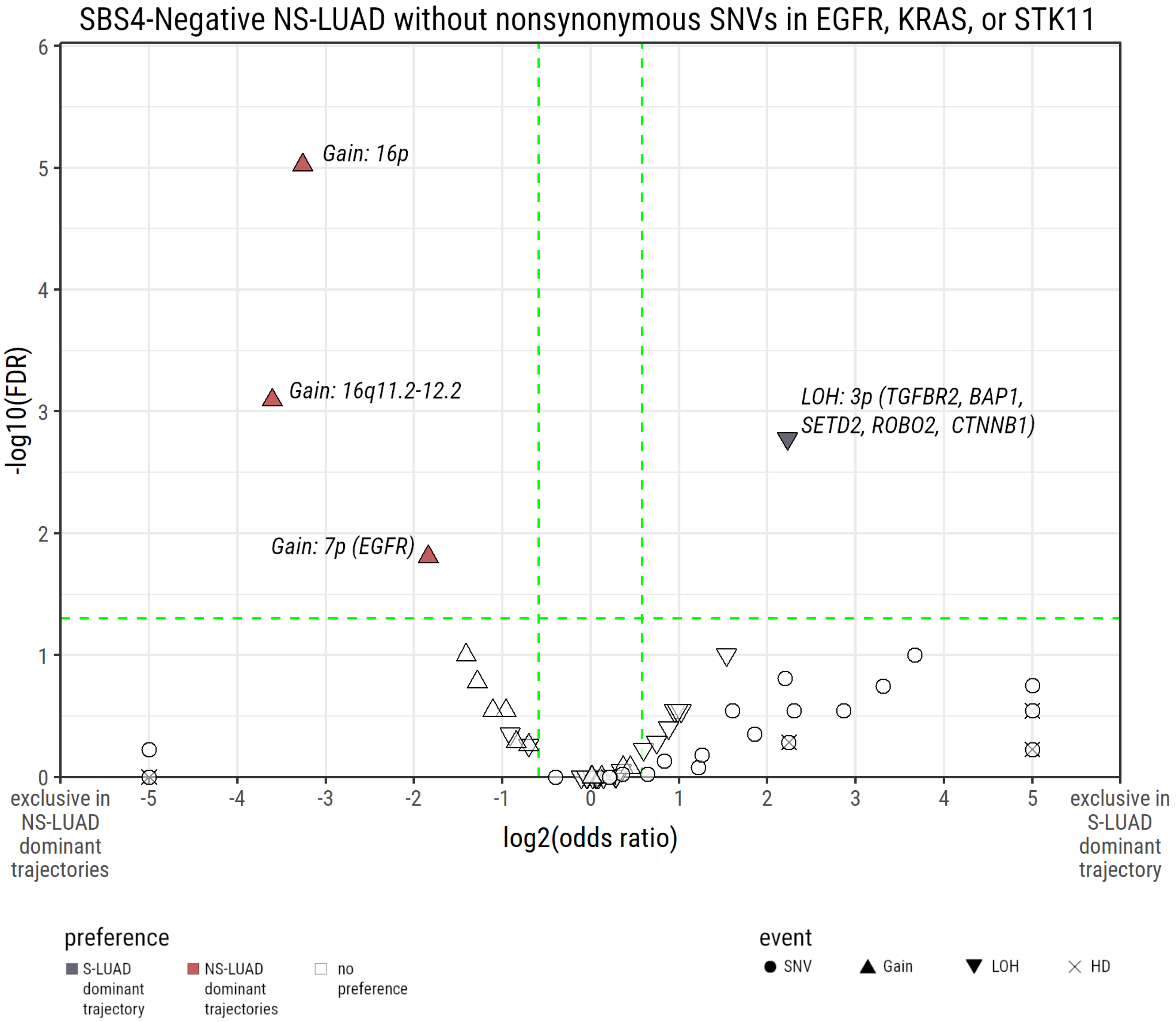

bioRxiv preprint doi: https://doi.org/10.64898/2025.12.19.695410; this version posted December 23, 2025. The copyright holder for this preprint (which was not certified by peer review) is the author/funder, who has granted bioRxiv a license to display the preprint in perpetuity. It is made available under aCC-BY 4.0 International license.

# Figure S9: Multicollinearity tests

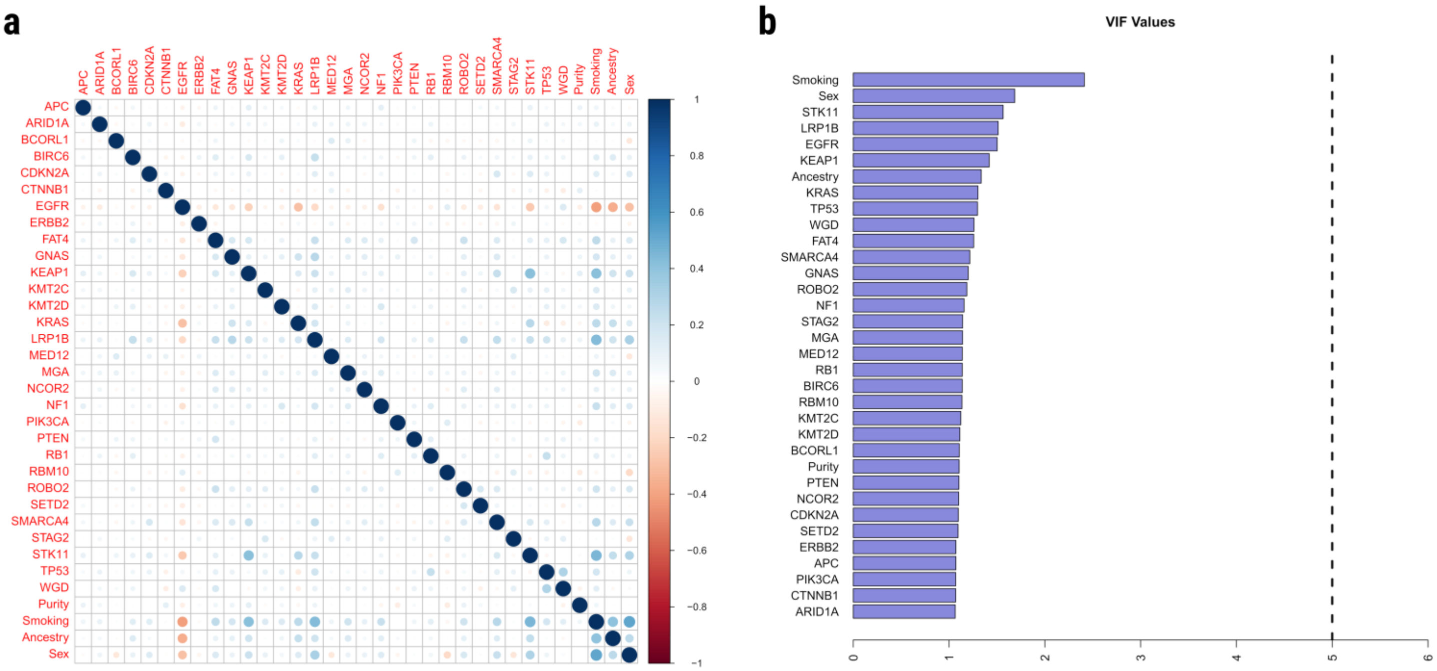

bioRxiv preprint doi: https://doi.org/10.64898/2025.12.19.695410; this version posted December 23, 2025. The copyright holder for this preprint (which was not certified by peer review) is the author/funder, who has granted bioRxiv a license to display the preprint in perpetuity. It is made available under aCC-BY 4.0 International license.

## Supplementary figure captions

**Figure S1: Selection of the optimal number of trajectories.** Boxplots showing the Bayesian Information Criterion (BIC) scores for 1000 iterations with each number of subsets from 1 to 5. 3 subsets were selected as optimal due to having the lowest median (BIC) value. Wilcoxon rank sum tests were performed on each pairwise comparison between numbers of subsets. Multiple testing corrected FDRs were $< 1x10^{-6}$ for each pairwise comparison.

**Figure S2: Event co-occurrence and mutual exclusivity.** Positive and negative associations between pairs of events across all LUAD, as calculated by hypergeometric distribution. Positive associations are shown in blue, negative associations in yellow, and lack of significant association shown in grey. Events with no significant positive or negative associations with any other event are excluded from the figure.

**Figure S3: Demographic distribution across trajectories.** Stacked bar plots showing the proportion of each demographic assigned to each trajectory. The proportion on the NSD-Loss trajectory is shown in blue, NSD-Gain in purple, and SD in grey. Statistical comparisons shown above barplots are two-tailed Fisher tests performed on the SD trajectory versus the combined NSD trajectories. Significant FDRs are written in green text. Comparisons shown are a) all LUAD split by self-reported smoking status, b) tumours in individuals of European ancestry split by self-reported smoking status (smoked vs never smoked), c) tumours in people who self-reported as having never smoked split by ancestry (European vs East Asian), d) tumours in people who self-reported as having never smoked split by SBS4 status (present vs absent), e) tumours in people who self-reported as having never smoked split by sex (male vs female), f) tumours in people who self-reported as having smoked split by sex (male vs female), and g) tumours in people who self-reported as having never smoked split by passive smoking exposure (yes vs no).

**Figure S4: Events showing a significant preference between S-LUAD and NS-LUAD.** A volcano plot showing the log2(odds ratio) and -log10(FDR) of event occurrence comparing tumours in people who self-reported as having smoked with those in people who self-reported as having never smoked. Events are coloured if odds ratio > 3/2 or < 2/3, and FDR < 0.05. Events associated with people who have smoked are coloured yellow, and events associated with people who have not smoked are coloured green. SNVs are represented by circles, copy number gains by upward pointed triangles, losses by downward pointed arrows, and HDs by crosses. Due to the number of events meeting the positive association criteria, events are only labelled if odds ratio > 8 or < 1/8, or if FDR $< 1^{-5}$.

**Figure S5: Major copy number of gains covering the MDM2 locus across different trajectories.** Boxplots show the major copy number in segments identified as gains, covering the locus of the MDM2 gene. Major copy number is shown for tumours on the NSD-Loss trajectory (blue, left), NSD-Gain trajectory (purple, centre), and SD trajectory (grey, right). FDRs shown relate to multiple testing corrected Wilcoxon tests between each pair of trajectories. Significant FDRs are shown in green.

**Figure S6: Mutational signatures showing a significant preference between trajectories.** Volcano plots show the log2(odds ratio) and -log10(FDR) of single base substitution (SBS), double base substitution (DBS), indel (ID), copy number (CN), and structural variant (SV) signature activity comparing: a) All LUAD between the SD ordering and the NSD-Loss & NSD-Gain orderings combined. b) NS-LUAD between the SD ordering and the NSD-Loss & NSD-Gain orderings combined. c) S-LUAD between the SD ordering and the NSD-Loss & NSD-Gain orderings combined. d) All LUAD between the NSD-Loss ordering and the NSD-Gain ordering. Signatures are coloured if odds ratio > 3/2 or < 2/3, and FDR < 0.05. Signatures are coloured according to their positive associations as follows: SD trajectory (vs NSD trajectories combined) - black, NSD trajectories combined (vs SD trajectory) - red, NSD-Loss (vs NSD-Gain) - blue, NSD-Gain (vs NSD-Loss) - purple, no association - white. SBS signatures are represented by circles, DBS signatures are represented by squares, ID signatures are represented by diamonds, CN signatures are represented by upward pointing triangles, and SV signatures are represented by downward pointing triangles.

**Figure S7: Adjusted survival curves by trajectory and smoking status.** Survival curves adjusted for age, sex, and tumour stage. a) shows survival of individuals of all smoking statuses, comparing between NSD-Loss, NSD-Gain, and SD trajectories. b) shows survival of NS-LUAD subjects, comparing between NSD-Loss, NSD-Gain, and SD trajectories. c) shows survival of all individuals, comparing people who self-reported as having smoked with people who self-reported as

bioRxiv preprint doi: https://doi.org/10.64898/2025.12.19.695410; this version posted December 23, 2025. The copyright holder for this preprint (which was not certified by peer review) is the author/funder, who has granted bioRxiv a license to display the preprint in perpetuity. It is made available under aCC-BY 4.0 International license.

having never smoked. Significance tests are adjusted curve tests - modified Pepe and Flemming tests for the difference between two adjusted survival curves - at 5 years. NSD-Loss curves are shown in blue, NSD-Gain curves are purple, SD curves are grey, the curve for those who have smoked is yellow, and the curve for those who have never smoked is green.

**Figure S8: NS-LUAD event preferences in the absence of *EGFR*, *KRAS*, or *STK11* mutations.** A volcano plot showing the log2(odds ratio) and -log10(FDR) of event occurrence comparing NS-LUAD between the *SD* ordering and the *NSD-Loss* & *NSD-Gain* orderings combined. Events are coloured if odds ratio > 3/2 or < 2/3, and FDR < 0.05. Events associated with the SD trajectory are coloured black and those associated with the NSD trajectories combined are coloured red. SNVs are represented by circles, copy number gains by upward pointed triangles, losses by downward pointed arrows, and HDs by crosses.

**Figure S9: Multicollinearity tests.** a) correlation between each pair of variables and b) variance inflation factor (VIF) of each variable to be included in multivariate linear analysis, in order to check that these variables were not subject to too high a degree of multicollinearity. In (a), positive correlations are shown in blue and negative correlations are shown in red. Larger correlations are represented both by darker colours and by larger circles. No pair of variables have a correlation score greater than 0.6. In (b), barplots show VIF values of each variable. A dashed line shows a threshold of 5. No variable has a VIF value greater than 3.
